# Isolated adrenocorticotropic hormone deficiency and sialadenitis associated with nivolumab: a case report

**DOI:** 10.1186/s13256-022-03663-6

**Published:** 2022-12-09

**Authors:** Sylvain Raoul Simeni Njonnou, Sandrine Aspeslagh, Marie-Josiane Ntsama Essomba, Marie-Lucie Racu, Fernando Kemta Lekpa, Frédéric Vandergheynst

**Affiliations:** 1 Department of Internal Medicine and Specialties, Faculty of Medicine and Pharmaceutical Sciences, University of Dschang, Dschang 96, Cameroon; 2 Department of Internal Medicine, Erasmus Hospital, Université Libre de Bruxelles, Route de Lennik 880, 1070 Brussels, Belgium; 3 Department of Medical Oncology, Erasmus Hospital, Université Libre de Bruxelles, Route de Lennik 880, 1070 Brussels, Belgium; 4 Department of Internal Medicine and Specialties, Faculty of Medicine and Biomedical Sciences, University of Yaounde I, Yaounde, Cameroon; 5 Department of Pathology, Erasmus Hospital, Université Libre de Bruxelles, Route de Lennik 880, 1070 Brussels, Belgium; 6 Dschang District Hospital, Dschang, Cameroon

**Keywords:** Sialadenitis, Isolated adrenal insufficiency (IAD), Hypophysitis, Brain methionine PET/MR, Aquaporins, Case report

## Abstract

**Background:**

Immune checkpoint inhibition with anti-PD(L)1 and anti-CTLA4 antibodies has significantly changed cancer treatment during the last 10 years. Nevertheless, boosting the immune system with immune checkpoint inhibition can result in immune-related adverse events, affecting different organ systems, among which the endocrine system is the most affected. However, there are few descriptions of the association of immune-related adverse events, and the pathophysiology of some is still lacking.

**Case summary:**

Here, we report a 70-year-old Caucasian patient treated with nivolumab (anti-PD1 monoclonal antibody) after resection of a unique relapse of melanoma in the neck region who presented with sicca syndrome, extreme fatigue, and weight loss 6 months after the start of anti-PD1 therapy. Blood tests revealed hypoglycemia and secondary hypocortisolism due to isolated adrenocorticotrophic hormone deficiency. Interestingly, brain methionine positron emission tomography/magnetic resonance revealed physiological metabolism of the pituitary gland, which was not increased in size, and no hypophyseal metastasis was detected. The sicca syndrome investigation revealed the absence of anti-SSA/SSB antibodies, while the labial salivary gland biopsy showed lymphoplasmatocytic infiltrates with a focus score of 1. To provide new insights into the physiopathology of the anti-PD1-related sialadenitis, we investigated the distribution of aquaporins 5 by immunostaining on the labial salivary gland acini, and compared this distribution with the one expressed in the primary Sjögren’s syndrome. Contrary to patients with primary Sjögren’s syndrome (in whom aquaporins 5 is mainly expressed at the basolateral side), but similar to the patients with no sialadenitis, we observed expression of aquaporins 5 at the apical pole. This new finding deserves to be confirmed in other patients with anti-PD1-related sialadenitis. Owing to these immune-related adverse events, anti-PD1 was stopped; nevertheless, the patient developed a new relapse 1 year later (March 2020) in the neck region, which was treated by radiotherapy. Since then, no relapse of melanoma was seen (1.5 years after radiotherapy), but the patient still requires hypophyseal replacement therapy. The sialoadenitis resolved partially.

**Conclusion:**

We report a combination of sialoadenitis and hypophysitis explaining extreme fatigue in a patient who was treated in the adjuvant setting with anti-PD1 for a melanoma relapse.

## Introduction

Immune checkpoint inhibitors (ICIs) represent a major advance in the treatment of many cancers [1, 2]. They improve survival in locally advanced and metastatic cancers by inhibiting the cytotoxic T lymphocyte antigens-4 (CTLA-4) and programmed death-1/programmed death ligand-1 (PD-1/PDL-1) [3, 4]. The CTLA-4 is a key receptor expressed on the surface of T lymphocytes, which transmits an inhibitory signal and ensures the breaking of the activation of T cells. The CTLA-4 blockage lifts the inhibitory signal and increases the activation of T cells [5, 6]. The activation of the PD1/PDL1 pathway downregulates the immune response for protecting healthy tissues [7].

Owing to the overstimulation of immune response and block of self-tolerance, immune-related adverse events (irAEs) can occur [8–12]. Endocrine diseases are among the main manifestation of irAEs. Their manifestations include thyroiditis, hypophysitis, or diabetes mellitus [13, 14]. Hypophysitis can be a life-threatening condition because of secondary adrenal insufficiency [15–19]. Sialadenitis, on the other hand, has an impact on quality of life [20–22]. The pathophysiology of endocrine manifestations has been widely explored, but sialadenitis physiopathology remains obscure. Recent work on the pathophysiology of primary Sjögren’s syndrome revealed that abnormal aquaporin (AQP) distribution and anti-AQP autoantibodies could jeopardize salivary secretion, but to the best of our knowledge, there is no work on either AQPs or AQP antibodies in ICI-associated sialadenitis [23, 24].

Here, we present a case of nivolumab-related sialadenitis and hypophysitis in a patient with melanoma who was referred to the internal medicine department for fatigue, sicca syndrome, and weight loss. Sicca syndrome improvement with corticosteroids is unusual in primary Sjögren’s syndrome (pSS). To compare this sialadenitis with pSS, we studied the distribution of AQP in the salivary gland of this patient.

## Case description

A 70-year-old Caucasian non-smoker man was referred to the internal medicine department with 3-month history of fatigue, anorexia, and sicca syndrome (xerostomia and xerophthalmia). He is a retired lecturer, living with his family in a warm social environment. He had no history of chronic illness or alcohol consumption and did not take any medicine. The patient was diagnosed with relapsing left cervical melanoma T4aN1aM0. He was treated by surgery (local resection of the tumor) and received nivolumab as adjuvant therapy. He started presenting fatigue, anorexia, and sicca syndrome after the eighth administration of nivolumab. The physical examination was unremarkable: blood pressure 112/86 mmHg, pulse 83 beats per minute, temperature 36.8 °C, and normal neurological examination. The ocular examination did not find keratitis, and Schirmer’s test was normal (7 mm left eye and 5 mm right eye). Unstimulated salivary flow (whole sialometry) was pathologic. Search for antinuclear antibodies (ANA) by anti-Sjögren’s syndrome A (SSA) and anti-Sjögren’s syndrome B (SSB), and was negative. Labial salivary gland (LSG) biopsy revealed focal lymphocytic infiltration with a Chisholm score of 3 (or focus score 1). The patient was treated with saliva substitutes and pilocarpine. Evolution was marked by a minor reduction of sicca syndrome, increased fatigue, anorexia, and weight loss.

**Fig. 1:**
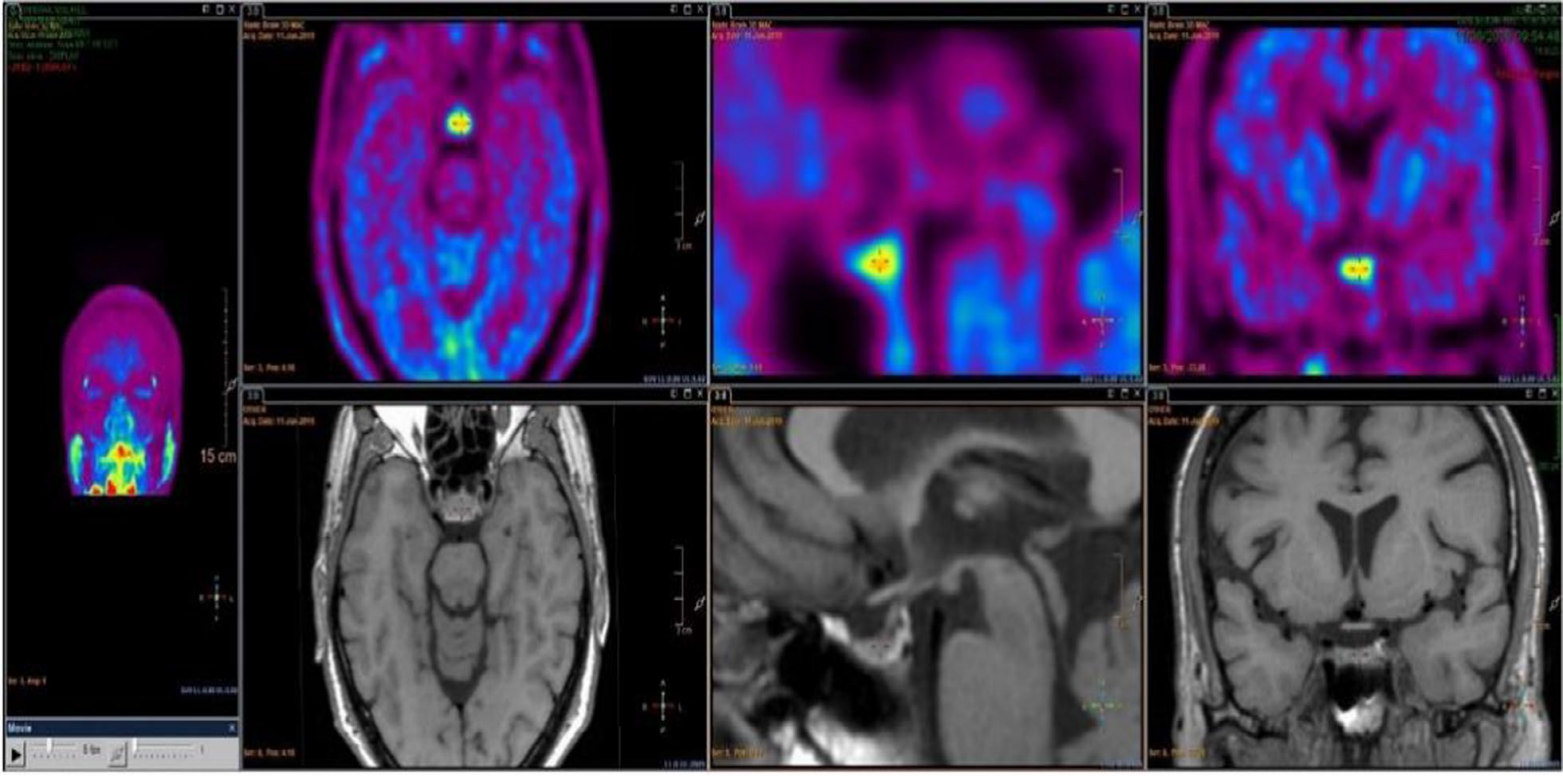
Methionine PET/MR showing no pituitary gland mass nor enlargement. Physiological metabolism of the pituitary gland

Persistent fatigue after a few weeks led to the suspicion of hypocortisolism. The 24-hour diuresis was normal, and there was no polyuria nor polidipsia syndrome. On biological investigation, there was an isolated adrenocorticotrophic hormone deficiency (IAD). Fasting blood sugar was 66 mg/dL (normal range 70–100 mg/dL). Cortisol was 26 nmol/L in the morning (normal range 166–507 nmol/L), and adrenocorticotropic hormone (ACTH) was 5.8 ng/L (normal range 7.2–63.3 ng/L). Normal gonadal and gonadotropic hormone levels were observed as follows: testosterone 17.8 ng/L (normal range 6.68–27.7 ng/L), luteinizing hormone (LH) 6.0 IU/L (normal range: 1.7–8.6 IU/L), and follicle-stimulating hormone (FSH) 8.4 IU/L (normal range 1.5–12.4 IU/L). Regarding the thyrotropic axis, thyroid-stimulating hormone (TSH) was 2.89 mU/L (normal range 0.3–4.2 mU/L); free tetraiodothyronine (T4), 18.8 pmol/L (normal range 12.0–22.0 pmol/L); and free triiodothyronine (T3), 3.8 pmol/L (normal range 3.1–6.8 pmol/L). Growth hormone (GH) was 16.7 mcg/L (normal range 12.0–22.0 mcg/L), and insulin-like growth factor-1 (IGF-1) was 127 mcg/L (normal range: 26–245 mcg/L). The natremia and kaliema were within the normal range, 140 mmol/L (normal range 136–145 mmol/L) and 3.9 mmol/L (normal range 3.5–4.5 mmol/L), respectively. Full blood count, urinalysis, and liver and renal tests were normal. No serology nor bacteriology test were performed. Table 1 summarizes the biological presentation at patient admission. To explore the metabolism of the pituitary gland and rule out any pituitary gland metastasis, an 11C-methionine positron emission tomography/magnetic resonance (methionine PET/MR) was performed. It was done 3 months after the beginning of symptoms and did not show any enlargement nor heterogeneity nor hypermetabolism of the pituitary gland (Fig. 1).

**Table 1:** Biological characteristics of the reported case at the time of onset of nivolumab-induced isolated adrenocorticotrophic hormone deficiency

Hormone	Patient’s value	Normal range
Morning AdrenoCorticoTropic Hormone (ACTH)	5.8 ng/L	8:00 am: 7.2–63 ng/L
Morning cortisol	26 nmol/L	8:00 am: 166–507 nmol/L
TSH	2.89 mU/L	0.3–4.20 mU/L
Free Tetraiodothyronine (fT4)	18.8 pmol/L	9.3–23.2 pmol/L
Free Triiodothyronine (fT3)	3.8 pmol/L	3.1–6.8 pmol/L
Luteinic Hormone (LH)	6.0 U/L	1.7–8.6 U/L
Follicular Stimulating Hormone (FSH)	8.4 U/L	1.5–12.4 U/L
Testosterone	17.8 ng/L	6.68–27.7 ng/L
C-Reactive Protein (CRP)	2.6 mg/L	< 5.0 mg/L
Fasting blood sugar	66 mg/dL	70–100 mg/dL
Insulin Growth factor-1 (IGF-1)	127 ng/mL	26–245 ng/mL
Growth hormone	16.7 mcg/L	12–22.0 mcg/L
Sodium	140 mmol/L	135–145 mmol/L
Urea	23.6 mg/dL	16.6–48.5 mg/dL
Serum creatinine	0.88 mg/dL	0.70–1.20 mg/dL
Total leukocytes	8.8 G/L	3.5–11.0 G/L
Neutrophil count	4.09 G/L	1.7–6.7 G/L
Lymphocytes count	3.68 G/L	1.2–3.5 G/L
Hemoglobin level	14.4 g/dL	13–18 g/dL
Platelet count	341 G/L	150–440 G/L
Alanine aminotransferase (GPT)	23 UI/L	< 41 UI/L
Aspartate aminotransferase (GOT)	18 UI/L	< 40 UI/L
Alkaline phosphatase	39 UI/L	40–129 UI/L
Gamma glutamyl transferase (GGT)	24 UI/L	10–71 UI/L
Urinalysis		
Protein	Negative	Negative
Blood	Negative	Negative
Density	1.020	1.005–1.020
pH	6	4.5–6.5

Given those results, a substitutive course of oral hydrocortisone (60 mg/day orally) was started. Evolution was therefore marked by a dramatic improvement of fatigue and anorexia, but only partial improvement of sicca syndrome. Hydrocortisone was tapered to 30 mg/day after 2 weeks. The patient presented a relapse in March 2020, 9 months after the diagnosis of hypophysitis. Given the adverse events with ICI, he was treated with radiotherapy. The last scan was performed in July 2021 without any sign of relapse.

## Pathological findings

**Fig. 2:**
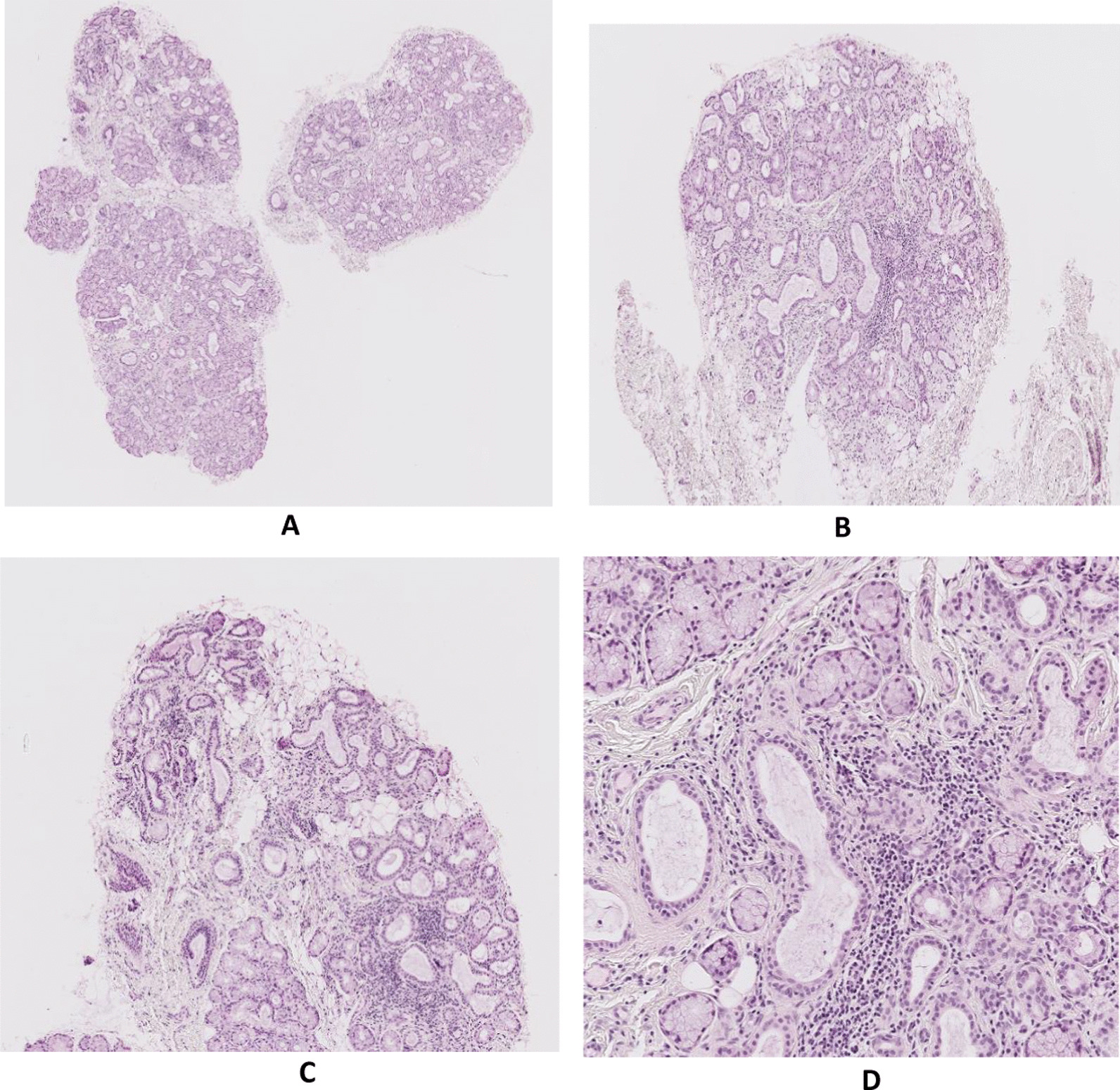
Hematoxylin–eosin staining of LSG showing lymphocytes infiltration and destruction of the gland (**A** ×2.5, **B** ×5, **C** ×10, **D** ×20)

**Fig. 3:**
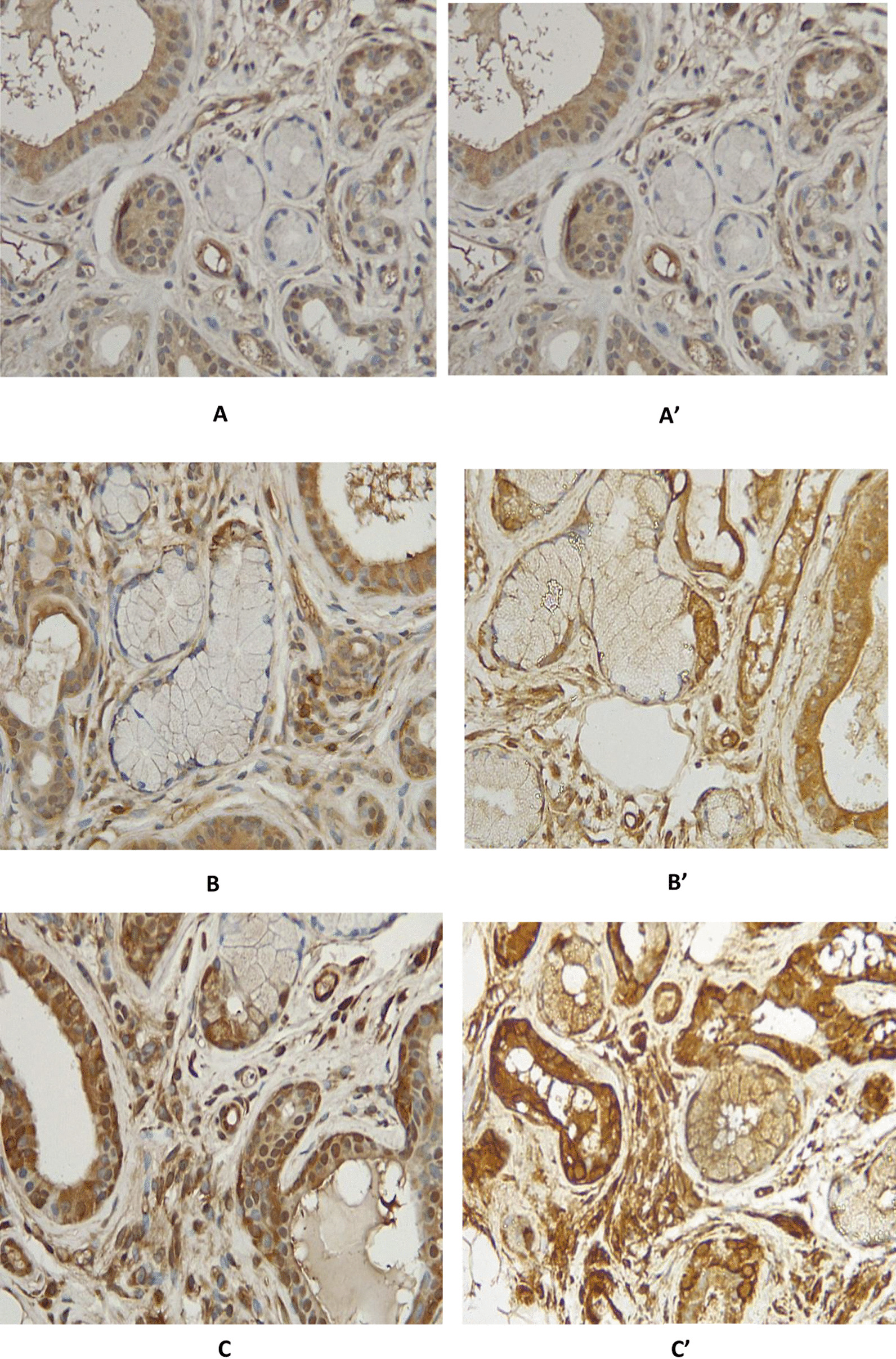
Immunostaining (×40) of AQP1 (**A** for the patient and **Aʹ** for healthy control), AQP3 (**B** for the patient and **Bʹ** for healthy control), and AQP5 (**C** for the patient and **Cʹ** for healthy control) showing no difference in the expression of these AQPs in the patient and control

Histological examination of the labial salivary gland revealed a focal lymphocytic sialadenitis with a Chisholm score of 3. There was no epithelitis (Fig. 2). Immunostaining on the LSG of the patient was performed and compared with that of the LSG of a healthy subject. Contrary to patients with pSS (in whom AQP5 is mainly expressed at the basolateral side), but similar to biopsies of patients with no signs of sicca syndrome [23, 24], we have observed expression of AQP5 at the apical pole (Fig. 3).

## Discussion

We describe the association of sialadenitis and an isolated adrenocorticotropic hormone deficiency (IAD) in a patient treated for relapsing locally advanced melanoma with nivolumab in the adjuvant setting. Nivolumab is an anti-PD-1 monoclonal antibody used as a treatment for many cancers, belonging to the class of ICI. The particularity of this case report belongs to: first, the association of sialadenitis and isolated adrenocorticotropic hormone deficiency, which has never been described before; second, the use of an 11C-methionine positron emission tomography/magnetic resonance (methionine PET/MR) for exploring the metabolism of the pituitary gland; and finally the immunostaining of aquaporins 1, 3, and 5 for understanding the pathophysiology of sialadenitis in this situation.

The endocrine system is the most commonly affected in the course of ICI treatment [25]. Type 1 diabetes mellitus, hypoparathyroidism, thyroid gland disorders, and hypothalamic–pituitary–adrenal axis (HPA) have been identified, with various prevalences according to the endocrine site and the molecule. Nivolumab was more associated with hypothyroidism than hypophysitis (with an incidence of 8% for hypothyroidism and 0.5% for hypophysitis in the metastatic setting) [14]. Hypophysitis has been described after administration of ICI. However, its prevalence varies according to detection means and diagnosis criteria (either clinical–radiological or biological). Otherwise, the hypophysitis prevalence and severity are higher with anti-CTLA4 than anti-PD(L)1. This difference may be due to CTL4 expression at the level of the hypophysis, so a different pathologic mechanism compared with the anti-PD1-induced hypophysitis [26]. The deficiency could be isolated (usually isolated adrenocorticotrophic hormone deficiency) or multiple (affecting mostly corticotropic, thyrotropic, and gonadotropic functions). Contrary to sporadic hypophysitis, which is more common in women, ICI-associated hypophysitis is two to five times more frequent in males than in females, and its occurrence increases with age (particularly with ipilumab) [14, 27]. Among patients with hypophysitis, isolated adrenocorticotrophic hormone deficiency (IAD) is the main presentation, as well as headache (particularly with anti-CTLA4) [27]. An autoimmune mechanism has been established, although in our case anti-pituitary antibodies were negative. However, hypophysitis is best explored by dynamic tests. Taieb Ach* et al*. reported the use of glucagon stimulation tests for the evaluation of the hypothalamic–hypopituitary–adrenal axis [28]. Radiologic abnormalities in hypophysitis, including pituitary gland enlargement with or without stalk thickening, are usually seen in the acute phase of hypophysitis, (more frequently with anti-CTLA4 than with anti-PD(L)1) [14]. This, with the delay of realization of brain MRI, might explain why no abnormalities were observed on our patient’s brain MRI.

There is no data on ICI-induced sialadenitis prevalence, although it is considered a very rare disease. Contrary to primary Sjögren’s syndrome (pSS), it is more frequent in men than women, with a higher mean age of occurrence [29]. ICI-associated sialadenitis presents these clues: sicca syndrome is common (mouth 96%, eye 65%, abnormal unstimulated salivary flow 86%) and autoantibodies (ANA+ in 52%, anti-SSA in 20%, rheumatoid factor in 9%) are less common than in patients with pSS [28]. Another difference between those two entities (pSS and ICI-associated sialadenitis) seems to be the therapeutic response to systemic corticosteroids, which is unusual in pSS [29]. The distribution of AQP5 on the immunostaining of LSG was similar to that of normal patients. This tends to prove that nivolumab-related sialadenitis is a very different clinical entity from pSS. Our patient had, however, a partial response to corticosteroids. The distribution of AQP5 on the immunostaining of LSG was similar to that of normal patients. This suggests that nivolumab-related sialadenitis is a different clinical entity from pSS.

Currently, to the best of our knowledge, there is no report of an association of isolated adrenocorticotrophic hormone deficiency with sialadenitis induced by ICI. An association of irAEs with a favorable oncological outcome is known, but their precise mechanism is still unclear [14, 30]. Specific HLA genotypes could be implicated in the occurrence of these irAEs as seen in patients who presented ICI-induced type 1 diabetes mellitus [31, 32].

## Conclusion

Persistent fatigue in a patient treated with ICI should be investigated to exclude hypophysitis (as well as radiologically and biologically). Similarly, the occurrence of sicca syndrome should lead to a thorough investigation. Whatever the side effect (hypophysitis or sialadenitis), an interruption of ICI treatment should be carried out and adequate treatment given. The physiopathology of sicca syndrome in patients with ICI needs to be confirmed in a larger sample of patients.

## Data Availability

The data that support the findings of this study are not publicly available owing to the respect of patient’s privacy but are available from SRSN (raoulsims@yahoo.fr) upon reasonable request.

## Abbreviations

ACTH: Adrenocorticotropic hormone
ANA: Antinuclear antibodies
AntiSSA: Anti-Sjögren’s syndrome A
Anti SSB: Anti-Sjögren’s syndrome B
AQP: Aquaporins
CTLA4: Cytotoxic T lymphocyte Antigen-4
FSH: Follicle-Stimulating Hormone
GH: Growth hormone
IAD: Isolated adrenocorticotropic hormone (ACTH) deficiency
ICI: Immune checkpoint inhibition/inhibitors
IrAE: Immune-related adverse events
LH: Luteinizing Hormone
LSG: Labial salivary gland
PD-1: Programmed death-1
PDL-1: Programmed death ligand-1
PET/MR: Positron emission tomography/magnetic resonance
pSS: Primary Sjögren’s syndrome
T3: Triiodothyronine
T4: Tetraiodothyronine
TSH: Thyroid-stimulating hormone
